# Cost-effectiveness of eye care services in Zambia

**DOI:** 10.1186/1478-7547-12-6

**Published:** 2014-02-25

**Authors:** Ulla K Griffiths, Fiammetta M Bozzani, Adrian Gheorghe, Lawrence Mwenge, Clare Gilbert

**Affiliations:** 1 Department of Global Health and Development, London School of Hygiene & Tropical Medicine, 15-17 Tavistock Place, London WC1H 9SH, UK; 2 ZAMBART Project, University of Zambia, Ridgeway Campus, Lusaka, Zambia; 3 International Centre for Eye Health, London School of Hygiene & Tropical Medicine, London, UK

**Keywords:** Costs, Cataract, Refractive error, Presbyopia, Quality of life

## Abstract

**Objective:**

To estimate the cost-effectiveness of cataract surgery and refractive error/presbyopia correction in Zambia.

**Methods:**

Primary data on costs and health related quality of life were collected in a prospective cohort study of 170 cataract and 113 refractive error/presbyopia patients recruited from three health facilities. Six months later, follow-up data were available from 77 and 41 patients who had received cataract surgery and spectacles, respectively. Costs were determined from patient interviews and micro-costing at the three health facilities. Utility values were gathered by administering the EQ-5D quality of life instrument immediately before and six months after cataract surgery or acquiring spectacles. A probabilistic state-transition model was used to generate cost-effectiveness estimates with uncertainty ranges.

**Results:**

Utility values significantly improved across the patient sample after cataract surgery and acquiring spectacles. Incremental costs per Quality Adjusted Life Years gained were US$ 259 for cataract surgery and US$ 375 for refractive error correction. The probabilities of the incremental cost-effectiveness ratios being below the Zambian gross national income per capita were 95% for both cataract surgery and refractive error correction.

**Conclusion:**

In spite of proven cost-effectiveness, severe health system constraints are likely to hamper scaling up of the interventions.

## Introduction

It is estimated that 39 million people were blind and 239 million visually impaired in 2010, with cataract and uncorrected refractive error (RE) being the leading causes [1]. This is also the case in southern Zambia, where a rapid assessment of avoidable blindness survey identified cataract (47%) and uncorrected RE (20%) as the principal causes of blindness [2]. However, a situation analysis of Zambian eye care services in 2011 concluded that there were major shortcomings, with lack of skilled human resources, inadequate spectacles manufacturing workshops and inequity in service provision between urban and rural areas [3].

The “Livingstone to Lusaka Urban Comprehensive Eye Care project” was implemented by Sightsavers between 2009–2014, with support from Standard Chartered Bank’s “Seeing is Believing” programme [4]. The aim of the project was to reduce blindness and ocular morbidity among approximately two million people living between Livingstone, in Southern Zambia, and the capital Lusaka. The objective of the present study was to estimate the cost-effectiveness of cataract surgery and RE/presbyopia correction delivered in three facilities supported by this project.

## Methods

Cataract surgery and correction of RE were chosen for this evaluation because the costs and health effects can be relatively well defined without long term follow-up. The study took a societal perspective, including costs incurred by both the health sector and patients. However, time costs of seeking care and productivity loss due to reduced eye sight were not included. This cost was excluded partly because cataract patients are generally older than the productive age and because of inherent problems of valuing time in settings with a large informal sector [5].

Data were collected between March and December 2011. Patient interviews were conducted by trained interviewers to collect socio-demographic data, information on Health Related Quality of Life (HRQOL) and out-of-pocket costs. Questionnaires and informed consent forms were translated and back-translated into the three main local languages. Information about the study was provided to participants orally in their own language. Signed consent was obtained when possible. Those who were illiterate provided a thumbprint. Patients were followed up at home six months after the intervention when the HRQOL instruments were re-administered. The cost and HRQOL data informed a decision analytic model for estimating the cost-effectiveness of cataract surgery and RE/presbyopia correction.

Ethical approval was granted by the University of Zambia and the London School of Hygiene & Tropical Medicine. Data were analysed in Microsoft Excel and STATA version 11.1.

### Study health facilities

Three health facilities were included. Lusaka Eye Hospital (LEH) is a private not-for-profit, urban tertiary facility with 40 beds, which offers advanced surgical procedures and employs at least one ophthalmologist at any one time [6]. Livingstone (semi-urban) and Choma (rural) General Hospitals are secondary level government facilities with eye units. Livingstone General Hospital has 325 beds; ten of which are for eye patients only. Choma General Hospital has 157 beds and eye patients are admitted to the general ward. Ophthalmic surgery is compromised at both these government facilities due to lack of skilled staff. At the time of the study, Livingstone had one ophthalmic clinical officer, one ophthalmic nurse and a part-time ophthalmologist, while Choma had one ophthalmic clinical officer and two general nurses. Surgeries in Choma were only performed during visits by ophthalmologists or cataract surgeons from other facilities. Services were free in the two Government facilities if patients were referred from a primary health facility and drugs were free if they were in stock at the hospital pharmacies. Patients not referred had to pay a registration fee of 30,000 Kwacha (US$ 6.34). Patients paid user fees at LEH, which covered the full costs of the service [6].

Cataract patients were recruited before surgery and those with RE/presbyopia were recruited immediately after their optometrist consultation. Patients who had the potential to benefit from spectacles according to the clinical judgement of the optometrist were included. At LEH and in Choma all patients were recruited at the hospital, but in Livingstone 57% of cataract and 66% of RE/presbyopia patients were recruited during outreach services undertaken as part of the Sightsavers project. At the two government hospitals, cataract patients were not operated immediately after baseline interviews as they had to wait for an ophthalmologist to be available.

### Sample size

In a Kenyan study the self-rated health Visual Analogue Scale (VAS) of the European Quality of Life Questionnaire (EQ-5D) was administered before and after cataract surgery [7]. Possible scores ranged from 0 (representing worst imaginable health state) to 100 (best imaginable health state). The mean score improved from 47.7 before surgery to 58.0 at follow-up [7]. To observe an improvement of the same magnitude with two-sided statistical significance of 1% and 90% power, 53 patients would be required. This was increased to 66 to account for 25% loss to follow-up, and we aimed for this sample size at all facilities.

A study on HRQOL improvement from correction of presbyopia in Zanzibar showed a 15-point difference in the general health dimension [8]. Hence, with 1% significance and 90% power, 17 patients per facility would be sufficient to detect an impact of correction with spectacles. The sample size was doubled to also include RE and further increased by 25% to account for loss to follow-up. The aim was thus to recruit 198 cataract patients and 126 RE/presbyopia patients across the three facilities.

### Visual acuity and quality of life measurement

Best-corrected visual acuity (VA) in the better-seeing eye was measured by the clinical staff at the facility using a Snellen chart and subsequently obtained from patient records. The Snellen values were converted into logMAR, by assigning a value of 1.85 logMAR to ‘counting fingers’ and 2.3 logMAR to vision of hand movements or less [9].

Changes in HRQOL due to cataract surgery and RE correction were measured from a survey that allowed for the calculation of utilities using EQ-5D [10]. This generic HRQOL instrument has five dimensions (mobility, self-care, usual activities, pain/discomfort and anxiety/depression) and each dimension has three levels (no problems, some problems or severe problems). The scale was designed by the European quality of life group to be brief, simple and easy to use alongside disease-specific measures [11]. The health states can be converted to a single utility value using a formula based on valuation from a general population sample [12]. Zimbabwe is the only African country where such population norms are available [13], and these were used in the present analysis; a choice justified because Zambia and Zimbabwe are neighbouring countries with similar cultures.

### Cost data collection and analysis

Mean costs per cataract surgery and RE/presbyopia correction were estimated by adding patient specific and facility overhead costs. Patient specific transport costs were collected from interviews, and types and quantities of drugs were gathered from patient records. Drug unit costs were obtained from hospital pharmacies. Facility costs specific to cataract surgery included medical equipment, surgical staff and supplies. Sightsavers procured polymethylmethacrylate intraocular lenses (IOL) at a unit cost of US$ 2.63 for Choma and Livingstone. LEH used the same IOLs, which were procured for US$ 6.00 per lens. Costs for RE/presbyopia correction included optometrist equipment, staff time and spectacles.

Since there was a spectacle manufacturing workshop at LEH, the mean costs of a pair of spectacles were estimated by dividing the total 2010 costs of this workshop by the annual number of spectacles produced. In Choma and Livingstone, the intention was that patients should be given spectacles at a subsidised price of 10,000–15,000 Kwacha (US$ 2–3), or free for those who could not afford it. Glasses were purchased by Sightsavers at US$ 4 per pair, including import duties. However, due to administrative problems in distributing the spectacles, some patients had to buy from optical shops. These costs were collected through interviews.

Costs of spectacles were not included for patients undergoing cataract surgery as in most African settings only a small proportion of cataract patients returns for follow up. Demand for near correction is also low as most patients are elderly, not literate and do not own or drive a car. Indeed, uncorrected refractive error is a common cause of visual impairment following cataract surgery [14].

Overhead costs were defined as all expenses that did not vary across patients, but were needed to run the health facility, such as administrative staff, buildings and utilities. Separate overhead costs were calculated for consultations and overnight stay in hospital. Recurrent costs were collected from 2010 expense records. Present values and life expectancies of capital costs, including buildings, vehicles and ophthalmology equipment, were approximated from procurement lists and by consulting staff in charge. The values of the Government buildings were collected from district Buildings Department offices. Capital items were amortised at a discount rate of 3% per year.

For the cost-effectiveness analysis, the mean costs across the three facilities were weighted according to the number of study patients recruited from each, to obtain an average cost for cataract surgery and spectacle provision. The range of costs between facilities was used to estimate standard deviations around mean values. Costs were estimated in 2010 US$ using an exchange rate of 4,729 Kwacha to one US$.

### State-transition modelling

**Figure 1:**
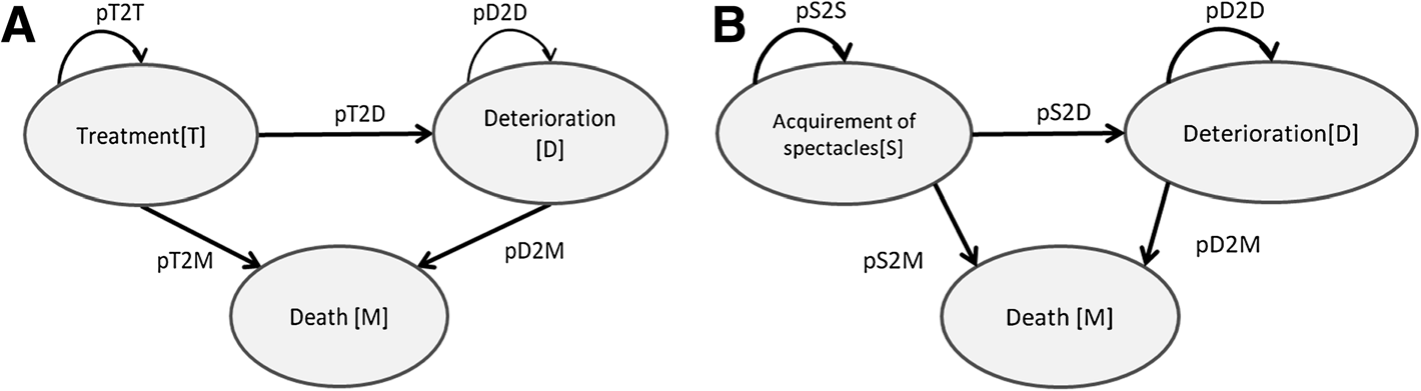
**Structure of the state-transition models. (A)** Cataract **(B)** Refractive error/presbyopia.

Two cohort state-transition models were developed to estimate the cost-effectiveness of eye care services compared to ‘no intervention’, expressed as incremental costs per Quality Adjusted Life Year (QALY) gained. The cataract surgery decision model (Figure 1A) assumed three health states: Treatment (T), deterioration (D) and death (M). Simulated patients entered the model at age 63 (the average age of the study sample) and were followed in annual cycles until death. After undergoing cataract surgery, all patients enter the T state and experience increased utility compared to baseline. To incorporate the possibility of ineffective or short-lived effects of surgery, it was assumed that at the end of each annual cycle, 4% of patients enter the D state, where baseline utility is experienced until death [15]. For the comparator cohort (no cataract surgery) all patients start in the D state.

The refraction model had a similar structure (Figure 1B), but the cohort was modelled from age 39 for five one-year cycles only. It was assumed that each year a proportion of patients will lose, break or damage their spectacles, with the probability increasing exponentially as a function of time from 5% at the end of the first cycle to 90% at the end of the five year modelling horizon. Future health effects were discounted by 3% per year and Zambian life tables were used to predict the annual probability of dying from all causes [16,17].

### Uncertainty and sensitivity analysis

Parameter uncertainty was incorporated by assigning probability distributions to costs, utilities and transition probabilities and running a Monte Carlo simulation with 10,000 iterations. Beta distributions were assigned to utilities and transition probabilities, and gamma distributions to costs [18]. The probability of the interventions being considered cost-effective was expressed as the probability of the incremental cost-effectiveness ratios being less than the 2011 Gross National Income (GNI) per capita in Zambia of US$ 1,160 [19]. This assumption was based on World Health Organization recommendations that an intervention with costs per Disability Adjusted Life Year averted of less than GNI per capita can be considered highly cost-effective [20]. It must however be emphasised that the Zambian Government has not established an official cost-effectiveness threshold.

A sensitivity analysis was undertaken by assuming the highest mean costs estimated from the three facilities.

## Results

### Recruitment and follow-up

**Figure 2:**
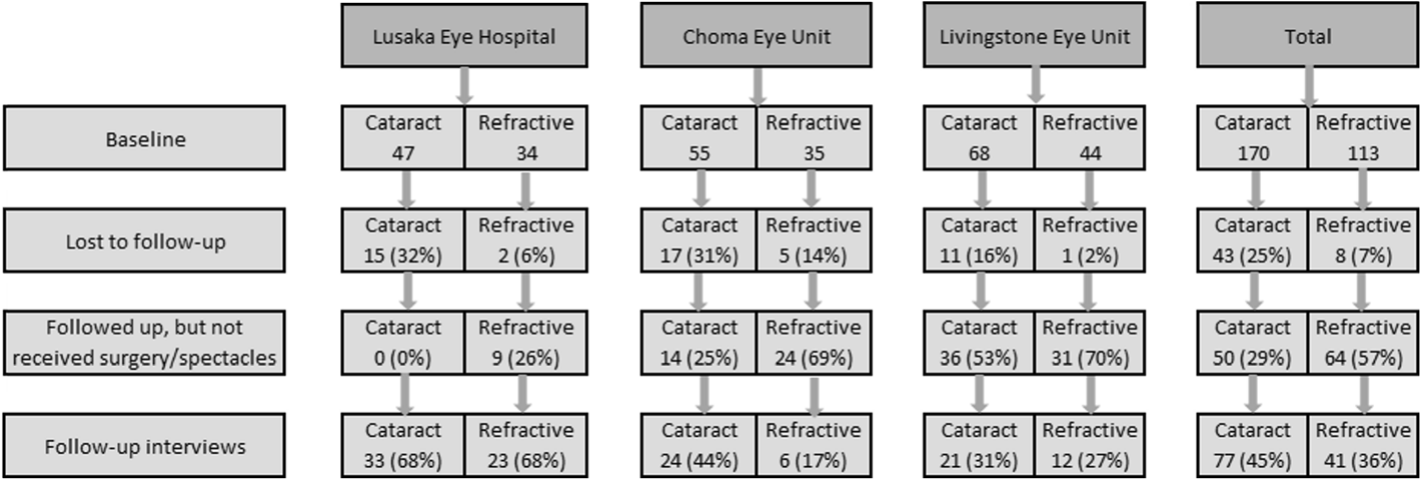
Patient flow in cohort study.

A total of 170 cataract and 113 RE/presbyopia patients were recruited at baseline (Figure 2). This was less than targeted due to irregular outreach services by Livingstone and Choma Hospitals. Loss to follow-up was 25% for cataract and 7% for RE/presbyopia patients. Cataract patients were difficult to follow-up as most were elderly and did not own a cell phone and, as they were no longer household heads, they were not known by name in their community. One patient from LEH was excluded because he was terminally ill at follow-up.

At follow-up, 29% of cataract patients had not received surgery, reasons being long waiting times (22%), being busy or not available at the time scheduled for surgery (20%), did not know where to go for surgery (18%), co-morbidities affecting the risk of surgery (8%), fear (8%), financial constraints (4%), and other or unclear reasons (18%). Fifty-seven percent of all patients who had been prescribed spectacles (49% of refractive errors and 70% of presbyopia) had not received them, with reasons being financial constraints (72%), long waiting times for free spectacles (16%), did not know where to collect (8%), and a belief that using glasses would damage the eyes (5%). The total number of patients included in the analysis was 77 for cataract, 28 for RE and 13 for presbyopia. Due to the low sample sizes, the RE and presbyobia patients were combined and we did not conduct separate analysis for the three facilities.

### Patient characteristics

Mean ages were 33, 50 and 63 years for RE, presbyopia and cataract patients, respectively (Table 1). Sixty two percent of presbyopia, 39% of RE and 8% of cataract patients had higher than secondary education. While the majority of interviews with RE and presbyopia patients were done in English, most cataract patients spoke local languages only.

**Table 1:** Patient socio-demographic characteristics

	**Cataract (n = 77)**	**Refractive error (n = 28)**	**Presbyopia (n = 13)**
**Percent female**	48%	64%	62%
**Age (years):**			
15–30	6 (8%)	15 (54%)	1 (8%)
31–50	10 (13%)	10 (36%)	4 (31%)
51–70	33 (43%)	2 (7%)	8 (62%)
70–91	28 (36%)	1 (4%)	0 (0%)
**Education**			
None	16 (21%)	4 (14%)	0 (0%)
Primary	36 (47%)	3 (11%)	0 (0%)
Secondary	19 (25%)	10 (36%)	5 (38%)
Higher	6 (8%)	11 (39%)	8 (62%)
**Marital status**			
Married	47 (61%)	10 (36%)	9 (69%)
Widowed	19 (25%)	1 (4%)	2 (15%)
Divorced	8 (10%)	3 (11%)	0 (0%)
Single	3 (4%)	14 (50%)	2 (15%)
**Language of interview**			
Nyanja	31 (40%)	6 (21%)	1 (8%)
Tonga	29 (38%)	1 (4%)	1 (8%)
English	17 (22%)	21 (75%)	11 (85%)

### Treatment costs

Mean treatment costs per cataract surgery were US$ 49, US$ 76 and US$ 111 at Choma, Livingstone and LEH, respectively, and the costs per RE correction were US$ 21, US$ 66 and US$ 70 at Choma, Livingstone and LEH, respectively (Table 2). Overhead costs at Choma were lower than at the two other facilities because of a relatively high volume of patients in spite of being a small facility. The total numbers of eye patients seen during 2010 were 17,413 at LEH, 3,039 at Livingstone and 2,682 at Choma.

**Table 2:** Mean costs per patient of cataract surgery and refractive error correction (2010 US$)

	**Lusaka eye hospital**		**Choma eye unit**		**Livingstone eye unit**	
	**Cataract surgery**	**Refraction**	**Cataract surgery**	**Refraction**	**Cataract surgery**	**Refraction**
General overhead	31	31	4	4	30	30
Surgery overhead	21	NA	9	NA	NC	NA
Diagnosis specific equipment	8	1	2	5	2	12
Diagnosis specific drugs and supplies	33	10	18	0.5	18	0.8
Diagnosis specific staff	18	12	16	7	26	3
Spectacles	NA	15	NA	4	NA	20
***Subtotal***	***111***	***70***	***49***	***21***	***76***	***66***
Transport	17	18	4	1	4	2
**Total**	**128**	**88**	**53**	**22**	**80**	**68**

NA: Not applicable, NC: not calculated.

The mean costs of spectacles were US$ 4 in Choma, US$ 15 at LEH and US$ 20 at Livingstone. In Choma all patients received their spectacles from the Sightsavers project, but in Livingstone five of the twelve patients bought their spectacles from an optical shop for between US$ 21 and US$ 106 per pair. At LEH, patients paid between US$ 12 and US$ 148 for spectacles, depending on the design, but the mean costs of production at the LEH manufacturing workshop were US$ 15. Mean, weighted costs across the three facilities used in the cost-effectiveness analysis were US$ 92 per cataract surgery and US$ 72 per RE correction (Table 3).

**Table 3:** Parameter values for the state-transition models

**Parameter**	**Mean (SE)**	**Distribution**	**Source**
**Costs (2010 US$)**			
Cataract surgery	92 (3.4)	Gamma	Cohort study
Corrective glasses	72 (7.8)	Gamma	Cohort study
**Utility values**			
Baseline cataract	0.782 (0.017)	Beta	Cohort study
Six months after cataract surgery	0.832 (0.015)	Beta	Cohort study
Baseline refractive error	0.850 (0.022)	Beta	Cohort study
Six months after refractive error correction	0.925 (0.018)	Beta	Cohort study
**Annual transition probabilities**			
Death	-	-	Zambia life tables
Deterioration after cataract surgery	0.04 (0.01)	Beta	Lundström and Wendel [15]
Deterioration after spectacles acquirement	0.05 (0.01)*	Beta	Assumed

*In the first year, then increasing exponentially each cycle so that 90% of the cohort has deteriorated spectacles by the end of the five-year time horizon.

### Transport costs

Mean travel time to the health facility one way was 35 minutes at Livingstone, 64 minutes at Choma and 77 minutes at LEH, ranging from less than 15 minutes to more than three hours for eight of the patients. At LEH, 51% used public transport, 45% their own car and 4% walked. At the two rural facilities, 20% used public transport, 19% their own car, and 61% walked. Transport costs were thus higher at LEH than at the rural facilities (Table 2).

### Visual acuity and utility values

All cataract patients were undergoing surgery to their first eye. In Zambia second eye surgery is uncommon on account of costs and low capacity of eye care services. Record keeping was sub-optimal, particularly at rural facilities, and reliable VA data were only available for 99 (35%) patients at baseline (52 for cataract, 41 for refractive error, and 6 for presbyopia), and only for 48 of the 77 cataract patients traced at follow-up. Mean logMAR VA (standard deviation) in the better-seeing eye at baseline was 0.24 (0.36) for RE, 0.17 (0.25) for presbyopia and 1.13 (0.72) for cataract. At first review one week after surgery, the mean VA of cataract patients was 1.12 (0.75).

There were significant differences in mean EQ-5D utility values between baseline and follow-up for both cataract and RE/presbyopia (Table 4), being 0.05 and 0.075 for cataract and RE, respectively. This level of change is comparable to impacts found for interventions to treat diseases such as arthritis and irritable bowel syndrome [21]. Cataract patients had lower utility values than RE/presbyopia patients at both baseline and follow-up.

**Table 4:** EQ-5D utility values at baseline and six months follow-up

	**Cataract (n = 77)**			**Refraction (n = 41)**		
**Utility value**	**Baseline**	**Follow-up**	P-value*	**Baseline**	**Follow-up**	P-value*
Mean	0.782	0.832	0.014	0.850	0.925	0.006
SD	0.150	0.129		0.139	0.117	
Min	0.130	0.417		0.361	0.509	
Max	1	1		1	1	

*P-value of a *t*-test for a difference in mean utility values between baseline and follow-up.

### Deterministic and probabilistic cost-effectiveness estimates

In the deterministic analysis, incremental costs per QALY gained were US$ 259 for cataract surgery and US$ 375 for RE/presbyopia correction (Table 5). When excluding transport costs, the costs per QALY gained were US$ 231 for cataract surgery and US$ 322 for RE/presbyopia correction. When using the mean costs of LEH in a sensitivity analysis, the incremental costs per QALY gained were US$ 360 for cataract surgery and US$ 458 for RE/presbyopia correction.

**Table 5:** Cost-effectiveness results (per patient over modelling horizon)

	**Cataract**			**Refractive error**		
	**Without surgery**	**With Surgery**	**Incremental**	**Without spectacles**	**With spectacles**	**Incremental**
**Deterministic analysis**						
Costs (2010 US$)	0	92	92	0	72	72
QALYs	7.519	7.874	0.355	3.655	3.847	0.192
Incremental costs per QALY gained			259			375
**Probabilistic analysis**						
Costs (2010 US$)	0	92	92	0	71	71
QALYs	7.519	7.876	0.356	3.654	3.750	0.096
Incremental costs per QALY gained			258			748

**Figure 3:**
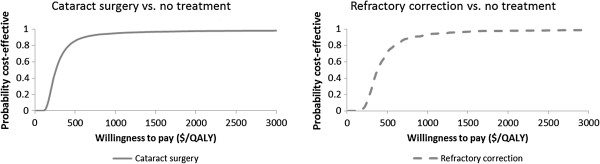
Cost-effectiveness acceptability curves derived from probabilistic uncertainty analysis.

The probabilities of the cost-effectiveness ratios being below the Zambian GNI per capita of US$ 1,160 were 95% for both cataract surgery and RE correction, as shown in the cost-effectiveness acceptability curves in Figure 3.

## Discussion

To our knowledge, this is the first economic evaluation of eye care interventions in Africa using primary data for costs as well as health effects. In the deterministic analysis, the costs per QALY gained were US$ 259 for cataract surgery and US$ 375 for RE/presbyopia correction.

We also believe that we are the first to elicit utility values from the Zambian population using a validated instrument. Other Zambian cost-effectiveness studies using QALYs as an outcome measure have focused on HIV/AIDS interventions [22-24] and used secondary utility data, such as from a meta-analysis of HIV utilities in high-income countries [25]. We showed that EQ-5D was sensitive to significant improvements in HRQOL due to the two interventions, which is noteworthy as EQ-5D is a generic instrument, which does not include dimensions specific to eye health. Previous studies have shown a lack of sensitivity to HRQOL changes for a range of eye conditions [26,27]. One reason for significant impact being detected in our study could be that patients in Zambia, similarly to other low-income settings, wait until they are virtually blind before they seek care [28]. This is not the case in high-income countries where cataract surgery and RE/presbyopia correction are provided earlier, so that visual acuity improvement and hence QALY gains will be relatively small. Our findings on the positive impact of cataract surgery on HRQOL in a low-income setting are consistent with findings from Kenya, the Philippines and Bangladesh [7].

The EQ-5D instrument used in this study has only three levels for each dimension. The EQ-5D-5L, which has five levels, has recently been developed to improve sensitivity. Evidence suggests that EQ-5D-5L has superior psychometric properties as it reduces the ceiling effect and has higher discriminatory power in patients with chronic diseases [29,30]. EQ-5D-5L may, therefore, capture more accurately the HRQOL benefits of cataract surgery and correction of REs.

Although evidence from a wide range of settings suggests that cataract surgery is generally cost-effective in both low- and high-income countries [31], the range in values is wide, i.e. US$ 1,486 per QALY gained in Japan [32] versus US$ 25,000 in the United Kingdom [33]. In a study from Tanzania costs per QALY gained from cataract surgery were estimated to be US$ 83 [31]. However, cost data in this study were informed by a willingness-to-pay approach [34] and utility values were sourced from high-income settings [35,36]. Few studies report the cost-effectiveness of RE correction. It has been suggested that screening and spectacles provision are highly cost-effective for school children [37,38] and estimates in the adult Australian population over a five-year horizon were approximately US$ 45 per QALY gained [39].

The worldwide variation in cost-effectiveness can be attributed to several factors. Firstly, the costs of cataract surgery vary. For example, a study of nine European countries reported costs of cataract surgery to vary from US$ 32,360 in Spain to $ 59,928 in Denmark, with health care labour duration, length of stay, inpatient vs. outpatient surgery and the type of IOL being the main cost determinants [40]. Secondly, lifetime estimates vary because the underlying decision models take markedly different approaches with respect to the modelled cohort and clinical pathways. For example, studies in the United Kingdom estimated the lifetime cost-effectiveness of first [41] and second-eye [33] cataract surgery by assuming only two health states (alive and dead) and constant lifetime surgery benefit. An argument can be made that factors such as the costs and incidence of complications of cataract surgery, visual acuity decline over time, and the patient response to visual acuity decline (e.g. immediate, delayed or no spectacles purchase) can and should also be taken into account [32,42]. However, implementing such considerations is always subject to the availability of data, especially in a context such as Zambia where few routine data are collected and eye health care services remain suboptimal [3].

The crisis in the health workforce in Africa impinged on our study in Zambia, as this was the primary reason why only 45% of cataract and 36% of RE/presbyopia patients received their expected services. Another reason could be that a proportion of our patients were recruited during outreach services. To receive cataract surgery or a spectacles prescription, the patients would have to travel to the health facility by their own means. During our follow-up interviews, it was revealed that patients found this difficult. Outreach services for eye health services are thus challenging and it is likely that the costs of successful provision of services in this manner are higher than the estimates presented in our study. A third constraint was the lack of spectacle manufacturing workshops outside Lusaka and an ineffective distribution system, which meant that patients had to wait an unreasonably long time for the spectacles to arrive.

The low uptake of services was a challenge to our cost estimates. We allocated overhead costs to all patients who attended the facilities, including those who were only screened, but did not receive the expected interventions. It can thus be argued that the costs per person effectively treated are higher than in our estimates, as a smaller number of patients would have to “share” the overhead costs. Moreover, establishment of an effective distribution system for spectacles would likely have increased the costs per pair of glasses. Estimating overhead costs in the two Government facilities was also difficult, partly due to non-computerised cost-accounting systems, but also because eye care constitutes a relatively small proportion of services, making it complex to determine what cost categories to include. A further study limitation was that costs of follow-up and treatment of complications after cataract surgery were not included. Since there were no standard procedures in place at the three facilities for patient follow-up, it was problematic to assess this cost. We do however recommend that future studies on the costs of cataract surgery make determined efforts to include this.

According to accepted international benchmarks, cataract surgery and RE/presbyopia correction can be considered highly cost-effective in Zambia [20]. However, severe health system and human resource constraints make it difficult to scale up services.

## Competing interests

All authors declare that they have no competing interests.

## Authors’ contributions

UKG conceived the study, supervised data collection, undertook the analysis and drafted the manuscript. FMB completed data collection, helped with the analysis and commented on the paper. AG completed the state-transition model and the probabilistic uncertainty analysis and provided inputs to the paper. LM helped with cost data collection. CG provided technical advice throughout the study. All authors read and approved the final manuscript.
